# Trends of tuberculosis meningitis and associated mortality in Texas, 2010-2017, a large population-based analysis

**DOI:** 10.1371/journal.pone.0212729

**Published:** 2019-02-28

**Authors:** Duc T. Nguyen, Saroochi Agarwal, Edward A. Graviss

**Affiliations:** Houston Methodist Research Institute, Houston, TX, United States of America; Universidad Nacional de la Plata, ARGENTINA

## Abstract

**Background:**

As the most severe form of tuberculosis (TB), TB meningitis (TBM) is still associated with high mortality even in developed countries. In certain areas of the United States (U.S.), more than 50% of the TBM patients die with TB or have neurological sequelae and complications despite the availability of advanced health care. This population-based analysis aimed to determine the risk factors and trends associated with TBM morbidity and mortality using state-wide surveillance data.

**Methods:**

De-identified surveillance data of all confirmed TB patients from the state of Texas between 01/2010 and 12/2017 reported to the National TB Surveillance System was analyzed. Spatial distribution of TBM cases was presented by Stata's Geographic Information Systems mapping. Univariate and multiple generalized linear modeling were used to identify risk factors associated with meningitis morbidity and mortality. Non-parametric testing was used to analyze morbidity and mortality trends.

**Results:**

Among 10,103 TB patients reported in Texas between 2010 and 2017, 192 (1.9%) had TBM. During this 8-year period, the TBM proportion fluctuated between 1.5% and 2.7% with peaks in 2011 (2.7%) and 2016 (2.1%) and an overall non-significant trend (z = -1.32, p = 0.19). TBM had a higher mortality at diagnosis (8.9%), during treatment (14.1%) and overall (22.9%) compared to non-TBM (1.9%, 5.3%, and 7.2%, respectively, p<0.001). While mortality during treatment was unchanged over time in non-TBM patients (z = 0.5, p = 0.62), it consistently increased in TBM patients after 2013 (z = 3.09, p = 0.002). TBM patients had nearly five times the risk for overall death in multivariate analysis [aRR 4.91 (95% CI 3.71, 6.51), p<0.001]. TBM patients were younger, and more likely to present with miliary TB or HIV (+). Age ≥45 years, resident of a long-term care facility, IDU, diabetes, chronic kidney disease, abnormal chest radiography, positive AFB smear or culture and HIV (+) were independently associated with higher mortality.

**Conclusion:**

TBM remains challenging in Texas with significantly high mortality. Risk factors determined by multivariate modeling will inform health professionals and lay a foundation for the development of more effective strategies for TBM prevention and management.

## Introduction

In 2017, an estimate of 6.4 million new tuberculosis (TB) patients were reported globally and 14% were diagnosed as extrapulmonary TB (EPTB) [1]. Tuberculous meningitis (TBM), a form of extrapulmonary TB and the most severe TB form, is an infection of the protective membranes (meninges) covering the central nervous system (CNS) by the *Mycobacterium tuberculosis* (*Mtb*). The diagnosis for TBM is clinically challenging and TBM treatment is difficult due to the poor blood–brain barrier penetrance of anti-TB medications [2]. The disease is associated with high mortality even in developed countries [3–5]. Despite the availability of advanced health care, more than 50% of TBM patients may have neurological sequelae and complications or die. More than two-thirds of the neurological complications occurred within the initial hospitalization and the mortality ranged from 19.3% to 21.5% [6, 7].

In the United States (U.S.), 84 TBM cases were identified in 2016, which accounted for 4.2% of 1,882 extrapulmonary TB cases reported and 0.9% of all the 9,272 confirmed TB patients [8]. A previous population-based study (Houston Tuberculosis Initiative, HTI) identified 108/4,204 (2.5%) cases of central nervous system TB (CNSTB) in Houston area between 1995 and 2004 [9]. Younger age, HIV infection and *Mtb* culture (-) were associated with CNSTB while older age, *Mtb* culture positivity (+) from a CNS source were associated with higher mortality. Compared with TB patients without CNS involvement, CNSTB patients were more likely to die within 180 days after TB diagnosis [9]. A more recent analysis using Texas surveillance data reported that TBM accounted for 7.5% of the population of exclusive EPTB [4]. Given the insufficiency of updated information on the trends and characteristics of TBM in settings with low TB prevalence, we conducted a population-based analysis using the TB surveillance data of the entire state of Texas to determine the trends and associated characteristics of the TBM morbidity and mortality, both overall and during TB treatment.

## Methods

### Study population

This analysis used the de-identified surveillance data of all confirmed TB patients from the state of Texas (TX) reported to the National TB Surveillance System (NTSS) between 01/2010 and 12/2017. The dataset was downloaded from the Centers for Disease Control and Prevention (CDC) supported TB Genotyping Information Management System (TBGIMS) website (https://www.cdc.gov/tb/programs/genotyping/tbgims/default.htm). TB meningitis was defined in the dataset as a binary variable (Yes/No) which indicates whether the site of TB disease involved the meninges or not [10]. Comorbidity data was recorded in the dataset from the patient’s medical chart or from the health care provider. Oral reporting or undocumented reporting from the patient or persons other than health care providers were not accepted [11]. *Mtb* culture conversion was defined when a patient had an initial positive TB culture that converted to a documented negative TB culture without converting back to a positive TB culture during the TB treatment course [10, 11].

### Ethics statement

As this was a retrospective study using de-identified secondary surveillance data, ethical approval was not required.

### Statistical analysis

Baseline characteristics were reported as frequencies and proportions. Differences in demographic and clinical characteristics between TBM and non-TBM patients were compared using the Chi-square or Fisher’s exact tests as appropriate. Stata's Geographic Information Systems (GIS) packet *spmap* (Stata Corp, LLC, College Station, TX, U.S.) was used to investigate the spatial distribution of TBM patients in different Texas counties. Non-parametric testing was used for the overall trends overtime for TBM morbidity and mortality.

Univariate and multiple generalized linear modeling (GLM) were used to determine the contribution of potential prognostic variables for TBM morbidity and patient outcomes. Risk ratio (RR) with 95% confidence interval (95% CI) was reported. Variables having a p-value of <0.2 in the univariate analysis or variables considered as clinically important were evaluated further in multiple GLM modeling. The Likelihood Ratio test was used to further reduce the model subsets. The best model was selected based on the smallest Bayesian information criterion (BIC). Model discrimination was determined by the area under the receiver operating characteristic (ROC) curve (AUC). Calibration of the model was determined by a non-significant Hosmer-Lemeshow’s goodness of fit test. All analyses were performed with Stata version 15.1 (StataCorp LLC, College Station, TX, USA). A p-value of <0.05 was considered statistically significant.

## Results

### Characteristics of the study sample

**Fig 1:**
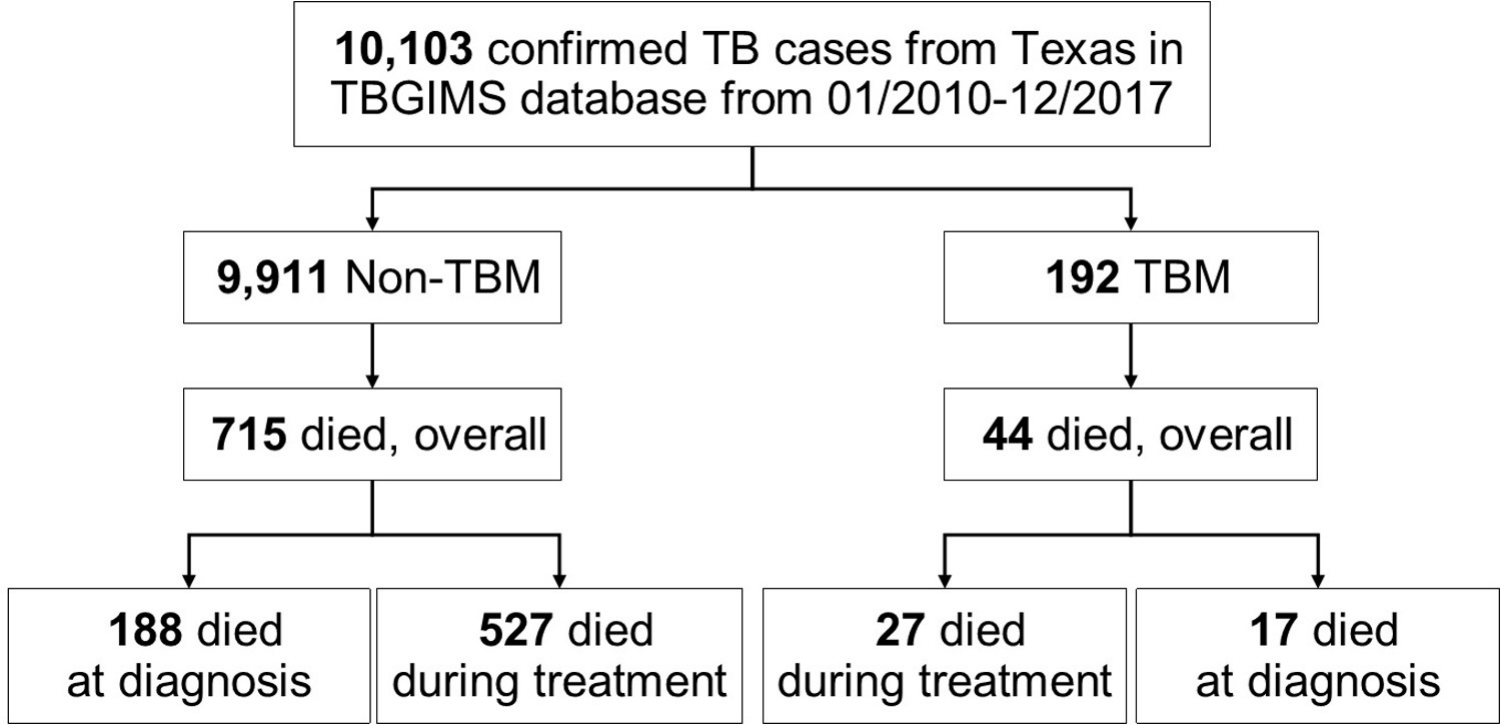
Flowchart of the study population. TBGIMS, Tuberculosis Genotyping Information Management System.

From January 2010 through December 2017, a total of 192 (1.9%) TBM patients were identified from 10,103 confirmed TB patients reported from Texas to the NTSS. In 44/192 (22.9%) of TBM patients who died, 61.4% (27/44) died during TB treatment and 38.4% (17/44) died at the time of diagnosis (prior to the TB treatment initiation). In the non-TBM group, 715/9,911 (7.2%) died, of whom, 527/715 (73.7%) died during the TB treatment and 188/715 (26.3%) died at time of diagnosis (Fig 1). Of 1,471 patients with exclusive EPTB, 113 (7.7%) TBM patients were identified. A total of 79/192 (41.2%) TBM patients were also diagnosed with pulmonary TB.

**Fig 2:**
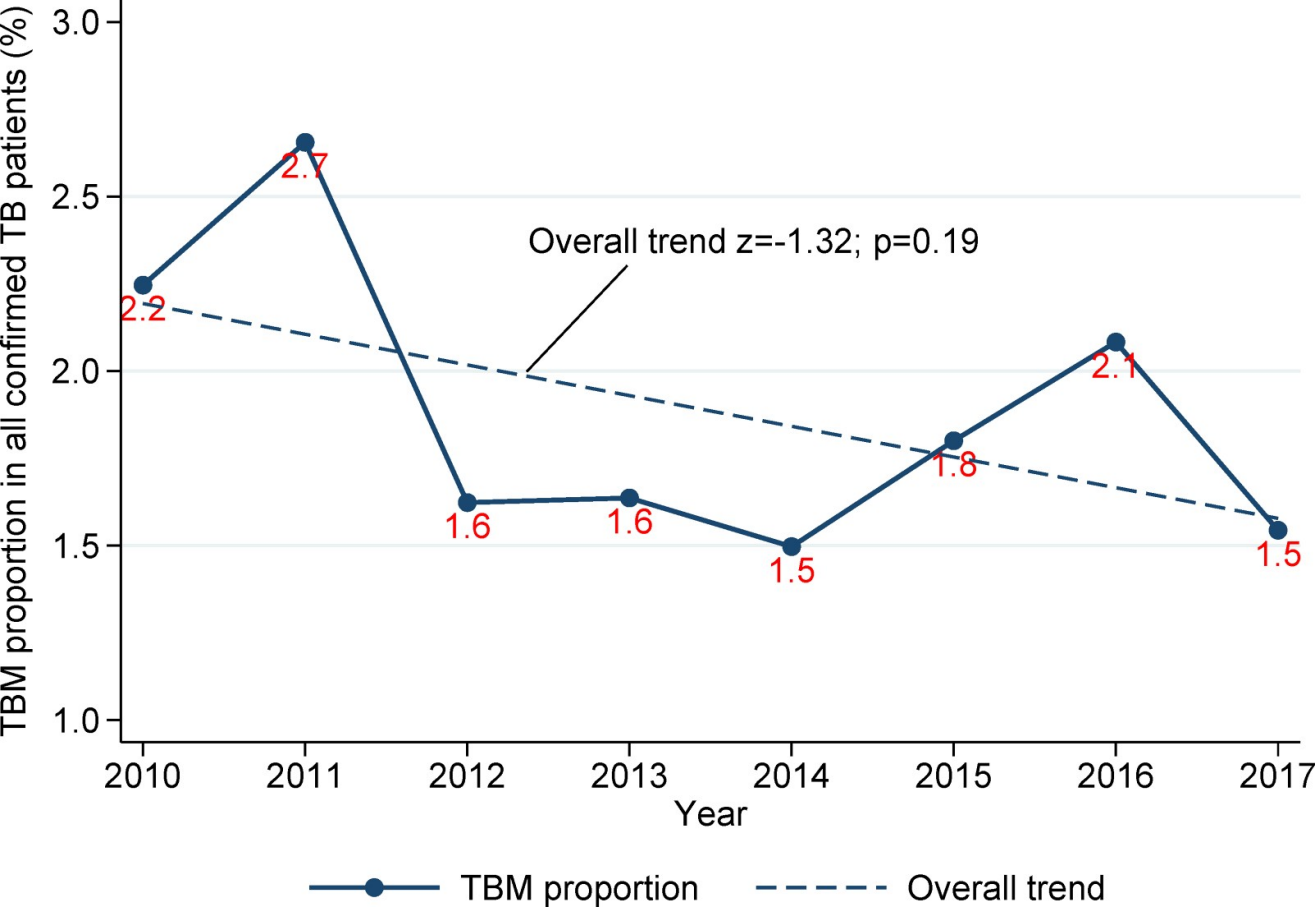
Overall trend of TB meningitis in confirmed TB patients in Texas, 01/2010-12/2017.

**Fig 3:**
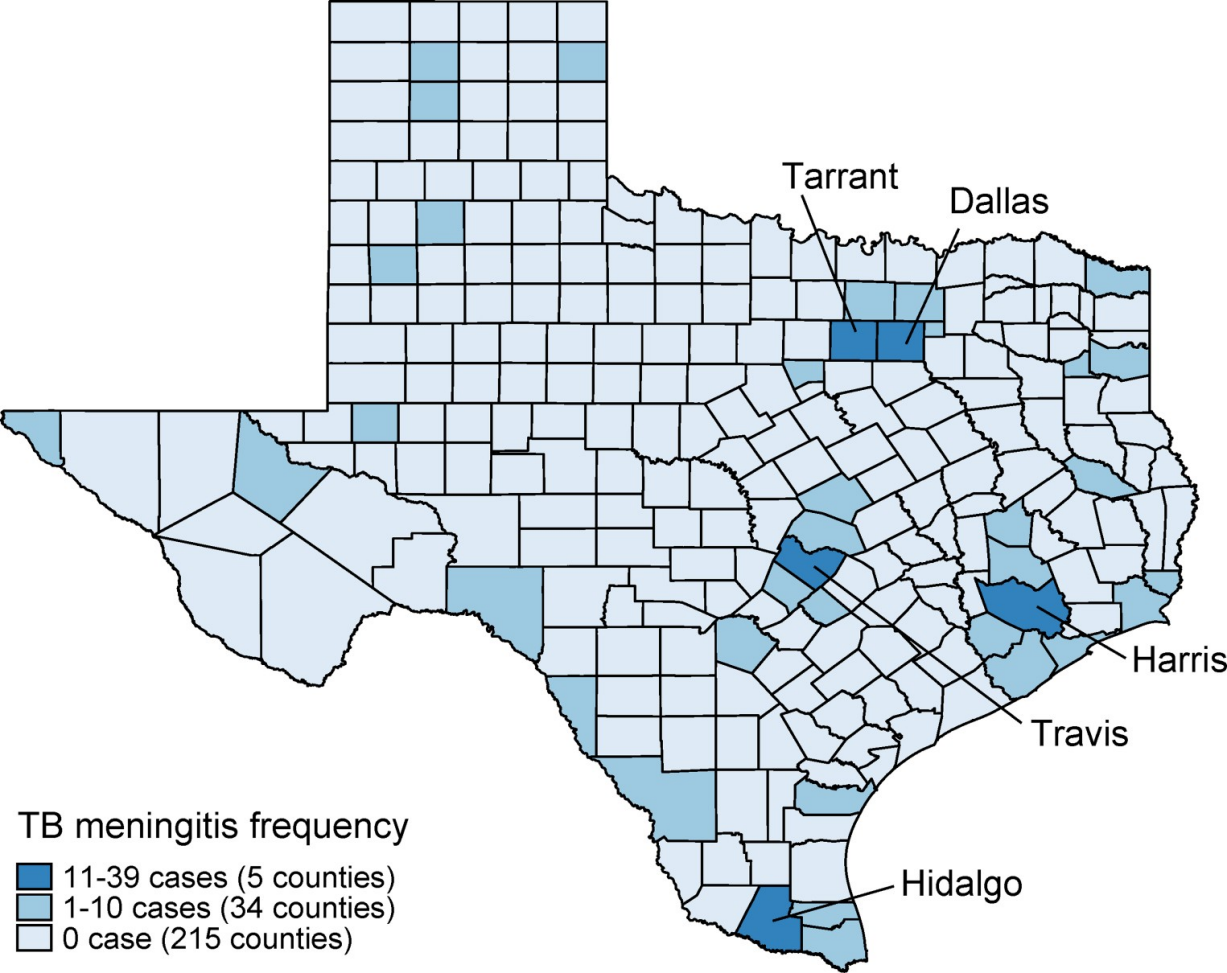
Spatial distribution of TB meningitis in Texas counties, 2010–2017.

The proportion of TBM was highest in the years 2011 (35/1,318; 2.7%) and 2016 (26/1,248; 2.1%). During the 8-year period from 2010 through 2017, the overall trend of TBM prevalence was not significantly different (z = -1.32; p = 0.19; Fig 2). In the exclusive EPTB patients, the proportion of TBM was 10.4% in 2010, increased to a peak of 11.5% in 2011 and gradually decreased to 7.7% in 2017 (overall trend z = -1.83; p = 0.07). GIS mapping of the distribution of TBM patients suggested a higher TB-DM burden in urban counties (Fig 3). The five Texas counties with the highest TBM prevalence were Travis (11/392; 2.8%), Dallas (37/1,445; 2.6%), Hidalgo (13/568, 2.3%), Tarrant (11/614; 1.8%) and Harris (40/2,411; 1.7%). Compared with the remaining counties in Texas, Hidalgo County (located on the Mexico-Texas border) had significantly higher frequencies of pediatric cases (below 15 years of age) both for all TB cases reported (57/568; 10.0% versus 579/9,535; 6.1%; p<0.001) and TBM (9/57; 15.8% versus 27/579; p = 0.001).

**Fig 4:**
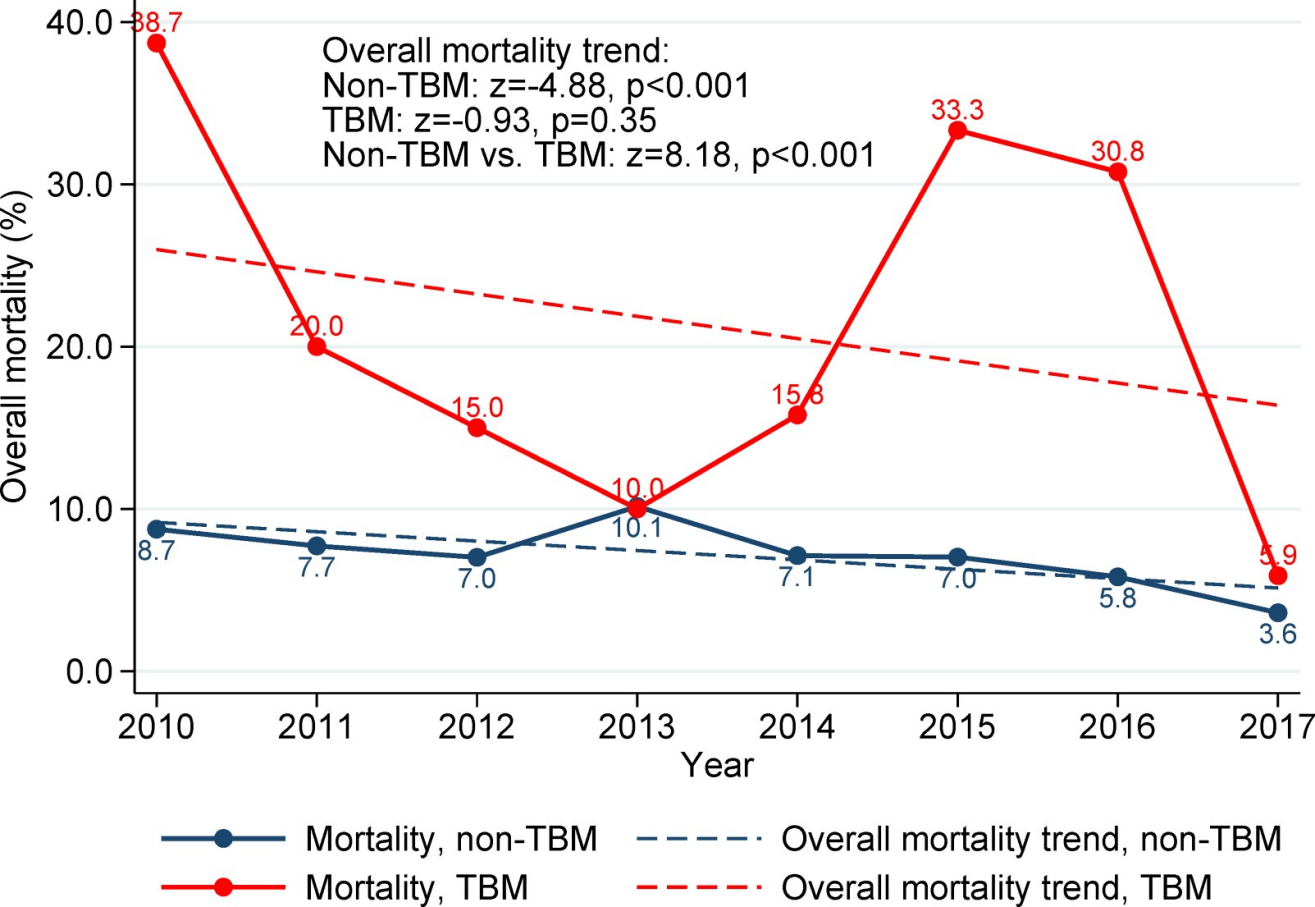
Trends of overall mortality in TB patients in Texas, 01/2010-12/2017, stratified by TB meningitis.

**Fig 5:**
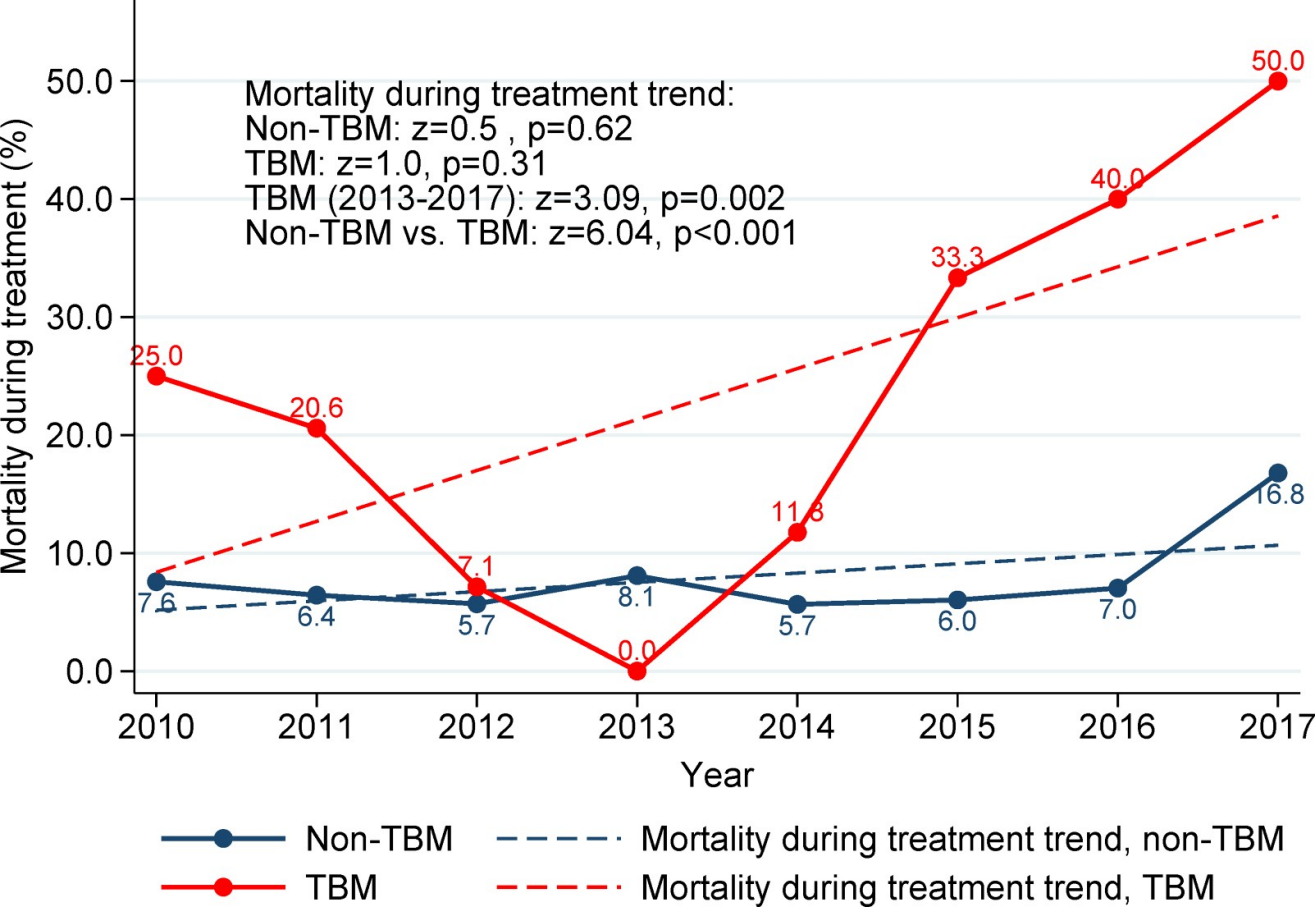
Trends of mortality during treatment in TB patients in Texas, 01/2010-12/2017, stratified by TB meningitis.

Patient characteristics and crude and adjusted associations with TBM are presented in Table 1. Compared with TB patients without meningitis, TBM patients were younger with a significantly higher proportion of children under <5 years of age (adjusted risk ratio [aRR] 3.39; 95% CI 1.83, 6.25; p<0.001), had a higher proportion of miliary TB (aRR 9.18, 95% CI 5.66, 14.88; p<0.001), and HIV co-infection (aRR 4.12; 95% CI 2.78, 6.10, p<0.001) (Table 1). The trends of overall mortality in confirmed TB patients are presented in Fig 4. From 2010 through 2017, non-TBM patients had a significant decrease in the overall mortality with an overall negative trend (z = -4.88, p<0.001), while the overall mortality of TBM patients, which was constantly and significantly higher than the overall mortality of non-TBM patients (z = 8.18; p<0.001), was not significantly different over time (z = -0.93; p = 0.35, Fig 4). The comparison of the mortality trends during TB treatment observed an upward trend from 2013 to 2017 in TBM patients (z = 3.09; p = 0.002) and this tendency was higher than the trend seen in non-TBM patients (z = 6.04; p<0.001) (Fig 5).

**Table 1:** Characteristics associated with TB meningitis in confirmed TB patients, Texas, 2010–2017 (N = 10,103).

	All	Non-meningitis	Meningitis	Unadjusted RR	Adjusted RR*	Adjusted
	(N = 10,103)	(*n* = 9,911)	(*n* = 192)	(95% CI)	(95% CI)	p-value*
Age (years)						
0–4	406 (4.0)	378 (3.8)	28 (14.6)	4.41 (2.46, 7.88)	3.39 (1.83, 6.25)	<0.001
5–14	230 (2.3)	222 (2.2)	8 (4.2)	2.22 (0.98, 5.05)	1.29 (0.54, 3.09)	0.56
15–24	1,150 (11.4)	1,132 (11.4)	18 (9.4)	(reference)	(reference)	
25–44	3,299 (32.7)	3,231 (32.6)	68 (35.4)	1.32 (0.79, 2.20)	0.83 (0.47, 1.46)	0.52
45–64	3,336 (33.0)	3,282 (33.1)	54 (28.1)	1.03 (0.61, 1.76)	0.86 (0.49, 1.53)	0.61
≥65	1,682 (16.6)	1,666 (16.8)	16 (8.3)	0.61 (0.31, 1.19)	0.71 (0.35, 1.45)	0.35
Male gender	6,585 (65.2)	6,473 (65.4)	112 (58.3)	0.75 (0.56, 0.99)		
White	1,136 (11.2)	1,123 (11.3)	13 (6.8)	0.57 (0.33, 1.00)		
U.S.-born	4,383 (50.4)	4,286 (50.3)	97 (56.7)	1.29 (0.96, 1.74)		
Homeless	543 (5.4)	538 (5.4)	5 (2.6)	0.47 (0.19, 1.14)	0.56 (0.21, 1.51)	0.26
Inmate of a correctional facility	1,017 (11.1)	1,010 (11.3)	7 (4.1)	0.34 (0.16, 0.72)	0.53 (0.24, 1.19)	0.12
Resident of long-term care facility	127 (1.3)	125 (1.3)	2 (1.0)	0.83 (0.21, 3.29)		
Injecting-drug user (IDU)	234 (2.3)	227 (2.3)	7 (3.6)	1.59 (0.76, 3.35)		
Non-IDU	980 (9.7)	965 (9.7)	15 (7.8)	0.79 (0.47, 1.33)		
Excessive alcohol use	1,713 (17.0)	1,691 (17.1)	22 (11.5)	0.64 (0.41, 0.99)		
Diabetes	1,606 (15.9)	1,590 (16.0)	16 (8.3)	0.48 (0.29, 0.80)		
Chronic kidney failure	150 (1.5)	147 (1.5)	3 (1.6)	1.05 (0.34, 3.26)		
Immunosuppression	180 (1.8)	172 (1.7)	8 (4.2)	2.40 (1.20, 4.79)		
Pulmonary TB	8,632 (85.4)	8,553 (86.3)	79 (41.1)	0.12 (0.09, 0.16)	0.14 (0.09, 0.21)	<0.001
TB of cervical lymph nodes	492 (4.9)	490 (4.9)	2 (1.0)	0.21 (0.05, 0.83)		
Miliary TB	264 (2.7)	242 (2.5)	22 (11.7)	4.83 (3.15, 7.40)	9.18 (5.66, 14.88)	<0.001
TB-CXR	8,392 (87.3)	8,302 (88.1)	90 (49.2)	0.14 (0.11, 0.19)	0.57 (0.39, 0.84)	0.010
Miliary TB on CXR	187 (2.2)	174 (2.1)	13 (14.4)	7.37 (4.17, 13.02)		
Cavitation on CXR	2,718 (32.4)	2,713 (32.7)	5 (5.6)	0.12 (0.05, 0.30)		
Positive AFB smear	3,702 (47.6)	3,689 (48.0)	13 (13.8)	0.18 (0.10, 0.32)		
Positive *Mtb c*ulture	5,515 (70.1)	5,485 (70.6)	30 (32.6)	0.21 (0.13, 0.32)		
Culture not converted (from + to -)	1,296 (12.8%)	1,285 (13.0%)	11 (5.7%)	2.07 (0.97, 4.40)		
Positive culture, NAAT, or AFB smear	7,848 (77.7)	7,726 (78.0)	122 (63.5)	0.50 (0.37, 0.67)	0.71 (0.52, 0.98)	0.04
Positive HIV status	642 (6.4)	605 (6.1)	37 (19.3)	3.52 (2.48, 4.99)	4.12 (2.78, 6.10)	<0.001
MDR-TB	70 (0.7)	67 (0.7)	3 (1.6)	2.90 (0.94, 8.93)		
East Asian *Mtb* family	1,210 (12.0)	1,192 (12.0)	18 (9.4)	1.02 (0.61, 1.69)		
Died at diagnosis	205 (2.0)	188 (1.9)	17 (8.9)	4.69 (2.91, 7.57)		
Died during TB treatment	554 (7.0)	527 (6.8)	27 (20.3)	3.37 (2.23, 5.09)		
Overall death	759 (7.5)	715 (7.2)	44 (22.9)	3.66 (2.64, 5.08)		

TB, tuberculosis; CXR, chest radiograph; TB-CXR, abnormalities on CXR consistent with tuberculosis; MDR-TB, multi-drug resistant TB; NAAT, nucleic acid amplification test; AFB, acid-fast bacilli.

*Adjusted in the multiple GLM model (area under the ROC curve, AUC = 0.87).

Multiple GLM modeling analyzing the association of all-cause mortality indicated that TBM patients had significantly higher mortality both at diagnosis (aRR 6.65; 95% CI 4.65, 9.52; p<0.001) and during TB treatment (aRR 4.29; 95% CI 2.93, 6.28; p<0.001). Generally, TBM patients had nearly a 5-fold increase in the risk for overall death compared with non-TBM patients. Age ≥45 years, long-term care facility residency, injecting drug user (IDU), diabetes, having chronic kidney failure, abnormalities on chest radiography consistent with tuberculosis (TB-CXR), miliary TB, TB confirmed by a positive culture, nucleic acid amplification test (NAAT) or AFB smear, and HIV co-infection were independently associated with a higher overall mortality. These characteristics were also significantly associated with mortality during TB treatment except for IDU and diabetes (Table 2). Stratified analysis by TBM status in patients having complete data for all the variables in the multivariate model indicated that although IDU remained insignificant in non-TBM patients, the variable became significant in TBM patients. Age ≥45 years and having a positive *Mtb* culture, nucleic acid amplification test or AFB smear were also significantly associated with higher mortality during TB treatment in TBM patients (Table 3).

**Table 2:** Characteristics associated with mortality in TB meningitis patients, Texas, 2010–2017.

	Overall mortality* (All patients, N = 9,569)		Died at diagnosis (All patients, N = 9,569)		Died during treatment (N = 7,530)**	
	Adjusted RR	Adjusted	Adjusted RR	Adjusted	Adjusted RR	Adjusted
	(95% CI)	p-value	(95% CI)	p-value	(95% CI)	p-value
TB meningitis	4.91 (3.71, 6.51)	<0.001	6.65 (4.65, 9.52)	<0.001	4.29 (2.93, 6.28)	<0.001
Age ≥45 (years)	5.91 (4.71, 7.42)	<0.001	5.95 (3.68, 9.62)	<0.001	5.89 (4.53, 7.64)	<0.001
Resident of long-term care facility	2.05 (1.54, 2.73)	<0.001	1.02 (0.47, 2.21)	0.96	2.33 (1.70, 3.20)	<0.001
IDU	1.55 (1.11, 2.17)	0.01	2.29 (1.23, 4.28)	0.01	1.39 (0.93, 2.08)	0.11
Diabetes	1.84 (1.02, 3.33)	0.040	1.46 (0.35, 6.09)	0.61	1.87 (0.97, 3.59)	0.06
Chronic kidney failure	3.35 (2.64, 4.25)	<0.001	2.39 (1.36, 4.20)	0.003	3.89 (2.97, 5.09)	<0.001
Miliary TB	2.12 (1.60, 2.81)	<0.001	1.61 (0.83, 3.13)	0.16	2.25 (1.65, 3.08)	<0.001
TB-CXR	1.69 (1.30, 2.18)	<0.001	1.01 (0.63, 1.61)	0.97	1.82 (1.31, 2.53)	<0.001
Positive TB culture, NAAT, or AFB smear	3.31 (2.46, 4.46)	<0.001	3.81 (1.95, 7.45)	<0.001	3.34 (2.41, 4.63)	<0.001
HIV status (+)	1.79 (1.39, 2.30)	<0.001	2.26 (1.25, 4.09)	0.01	1.81 (1.36, 2.40)	<0.001
Diabetes*age (interaction term)	0.43 (0.23, 0.79)	0.01	0.52 (0.12, 2.30)	0.39	0.43 (0.22, 0.84)	0.01

Values are in number (%);

*Including patients with unknown vital status;

**Including only patients having treatment outcomes as "Died" or "Completed";

CXR: chest radiograph; TB-CXR, abnormalities on CXR consistent with TB; MDR-TB, Multi-drug resistant TB; NAAT, nucleic acid amplification test; Models were run on patients who have data available for all variables in the model; RR, risk ratio. The area under the ROC curve of the multivariate models for overall mortality, died at diagnosis and died during treatment was 0.82, 0.81 and 0.81, respectively.

**Table 3:** Adjusted association of characteristics and mortality during TB treatment, stratified by meningitis status in patients having complete data.

	Non-meningitis (*n* = 7,746)		Meningitis (*n* = 133)	
	Adjusted RR	Adjusted	Adjusted RR	Adjusted
	(95% CI)	p-value	(95% CI)	p-value
Age ≥45 (years)	6.07 (4.59, 8.01)	<0.001	3.78 (1.72, 8.33)	0.001
Resident of long-term care facility	2.32 (1.68, 3.19)	<0.001	3.26 (0.65, 16.43)	0.15
IDU	1.29 (0.82, 2.03)	0.27	2.74 (0.92, 8.16)	0.070
Diabetes	1.93 (1.00, 3.74)	0.051	1.19 (0.42, 3.35)	0.74
Chronic kidney failure	3.94 (3.00, 5.18)	<0.001	—	—
Miliary TB	2.45 (1.78, 3.37)	<0.001	1.16 (0.46, 2.95)	0.75
TB-CXR	2.10 (1.42, 3.10)	<0.001	1.39 (0.70, 2.78)	0.35
Positive culture, NAAT, or AFB smear	3.41 (2.40, 4.85)	<0.001	3.33 (1.21, 9.17)	0.02
Positive HIV status	1.89 (1.41, 2.54)	<0.001	0.89 (0.40, 2.01)	0.79
Diabetes*age (interaction term)	0.40 (0.20, 0.80)	0.01	—	—

Analysis included only patients having treatment outcomes as "Died" or "Completed"; CXR: chest radiograph; TB-CXR, abnormalities on CXR consistent with tuberculosis; MDR-TB, Multi-drug resistant TB; NAAT, nucleic acid amplification test; Models were run on patients who have data available for all variables in the model. The area under the ROC curve of the multivariate models run in non-meningitis and meningitis patients was 0.80 and 0.72, respectively.

## Discussion

Our analysis using state-wide surveillance data found a significantly higher proportion of TBM in Texas compared with the national average [8]. From 2010 through 2017, no significant decrease was found in the trend of TBM in Texas. While the overall mortality in non-TBM patients significantly decreased over time from 2010 through 2017, trend analysis did not observe a similar trend in the cohort of TBM patients. Importantly, mortality in TBM patients was consistently higher compared with non-TBM patients during the 8 years of surveillance data analyzed.

Multiple GLM modeling indicated that TBM patients had significantly poorer outcomes compared with TB patients without meningitis with more than nine, twelve and seven times the odds for overall mortality, mortality prior to the initiation of TB treatment and mortality during treatment, respectively. Our findings confirm the tremendous challenges in managing TBM patients even in settings with advanced health care systems and with the availability of well-deployed local and state TB control programs [6, 12]. Older age, IDU, diabetes, chronic kidney failure, military TB, TB-CXR, positive *Mtb* culture and HIV infection were independently associated with higher overall mortality. These finds are consistent with observations reported by other authors in high income countries [13–16].

The demographic and clinical risk factors identified by multivariate modeling in this analyses can be used to develop prognostic scoring systems that use the routinely-collected clinical characteristics to predict patient outcomes earlier as suggested by previous work, especially in settings of low TB prevalence [17–19]. Analysis of mortality during treatment suggested that older age, IDU and a positive *Mtb* culture remained significant after stratifying by TBM status. Although HIV infection has been recognized as a significant risk factor for mortality during TB treatment [17, 20], our stratified analysis found HIV infection was only significant in the non-TBM population, but appears not to have enough power to be identified as a significant risk factor in TBM patient.

Our multivariate analysis also identified the population subgroups that were associated with TBM morbidity (younger age, miliary TB and HIV infection). These risks, especially HIV infection, have been recognized as being associated with TBM [20–23]. A higher cerebrospinal fluid HIV-1 viral load has been observed in TBM patients in the setting of high TB and HIV infection prevalence [24]. However, immediate antiretroviral treatment initiation has been reported not to improve outcomes in TBM patients [25].

It is not surprising that urban Texas counties such as Travis, Dallas, Tarrant and Harris were identified with the highest frequencies of TBM cases as they also have the highest frequency of TB cases [26]. Interestingly, we also found a high frequency of TBM in Hidalgo, a county locating adjacent to the Mexican border. The high prevalence of TB cases in Hidalgo and counties along the Mexican border have been recognized before, probably due to the migration flow from Mexico to Texas [27, 28]. Given that Hidalgo County has significantly higher frequencies of pediatric cases for both TB and TBM cases compared with other counties in our analysis, the transient border crossing of non-US residents from Mexico to Texas may play a role. However, this issue is not within the scope of this paper.

Our study has some notable limitations. First, although we identified a higher proportion of the TBM cases than the U.S. national average, the sample size may not have enough power to detect certain potential risk factors. Second, given the nature of surveillance data where certain historical information was obtained from patient interviews, recall bias cannot be completely ruled out. Thirdly, treatment time and time to event data were not available and prevent us from performing more robust survival analyses. Additionally, clinical data regarding cerebrospinal fluid (CSF) results and neurologic examination were also not available, which prevented us from developing more robust predictive models. However, the current multivariate models still have strong discrimination power with an area under the ROC curve of 0.87 (for TBM morbidity) or 0.82 (for overall mortality). Lastly, our results may not be generalizable to other state populations of other states without further validation given the diversity and TB prevalence of Texas.

## Conclusion

TBM remains a challenge in Texas with significantly higher mortality despite the availability of advanced health care facilities. The study’s findings confirm the need for more extensive studies to inform clinicians and other health professionals, as well as, develop more practical algorithms to promptly identify patients with higher risks for mortality and effectively manage TBM patients after identification.
